# Evidence for object–place binding in pigeons in a sequence-learning procedure

**DOI:** 10.3758/s13420-022-00521-9

**Published:** 2022-04-04

**Authors:** Aaron P. Blaisdell, Julia E. Schroeder

**Affiliations:** UCLA Department of Psychology, University of California, 1285 Franz Hall, Los Angeles, CA 90095-1563 USA

**Keywords:** Pigeons, Binding, Sequence learning

## Abstract

We studied object–location binding in pigeons using a sequence learning procedure. A sequence of four objects was presented, one at a time at one of four locations on a touchscreen. A single peck at the object ended the trial, and food reinforcement was delivered intermittently. In Experiment 1, a between-subjects design was used to present objects, locations, or both in a regular sequence or randomly. Response time costs on nonreinforced probe tests on which object order, location order, or both were disrupted revealed sequence learning effects. Pigeons encoded location order when it was consistent, but not object order when it alone was consistent. When both were consistent, pigeons encoded both, and showed evidence of object–location binding. In Experiment 2, two groups of pigeons received training on sequences where the same object always appeared at the same location. For some pigeons a consistent sequence was used while for others sequence order was randomized. Only when sequence order was consistent was object–location binding found. These experiments are the first demonstrations of strong and lasting feature binding in pigeons and are consistent with a functional account of learning.

When we open our eyes, we see a world populated by objects. Objects appear as coherent sets of features and properties that become bound together in perception. There has been a prodigious amount of work investigating perceptual binding since the seminal work by Anne Treisman and her associates (Treisman & Gelade, 1980). One emergent finding from this literature is that attention is a necessary driver of binding. Experiments performed in monkeys (Luck et al., 1997) and humans (Kastner et al., 1998) have identified common neural mechanisms of attention that modulate perceptual binding. Moreover, attention appears to play an important role in the storage of feature-biding in memory—what we will refer to as “memory feature biding” to contrast it to “perceptual feature binding.” We believe both perceptual and memory feature binding involve the same perceptual binding mechanisms but are shown under different conditions and using different procedures. Specifically, perceptual feature binding is demonstrated in tasks that show an immediate effect during stimulus presentation and encoding. Memory feature binding, on the other hand, is typically shown on a memory test for prior learning, or after a lot of practice, such as on a Serial Reaction Time (SRT) procedure, and likely reflects the outcome of associative processes that bind contiguous perceptual features into a memory engram. Given the similar role of attentional processes in binding in both monkeys and humans, this raises the question of how broadly across the animal kingdom does attention modulate binding? Does binding in other species require attentional processes? We investigated this question by studying the role of attention in binding in pigeons.

Pigeons are an ideal animal model in which to study this question. There is much known about the learning, memory, and perceptual capacities of pigeons, which serve as one of the most common nonmammalian models in psychology (Cook, 2001). The avian visual system has evolved functional homologies to the primate visual system in the ability to rapidly analyze visual information (e.g., Cook, 2000; Husband & Shimizu, 2001; Wylie et al., 2009). Furthermore, pigeons show many of the same attentional (Blough, 1991; Blough & Blough, 1997; Cook et al., 2012) and perceptual (Cook, 1992a, 1992b) processes necessary to support binding. Despite these homologies, the few studies that have directly looked for perceptual binding in pigeons have found weak or no evidence (Katz et al., 2010; Lazareva & Wasserman, 2016). Lazareva and Wasserman (2016) used a change-detection task in which, on each trial, pigeons were presented with two consecutive multi-item displays. Pigeons were reinforced for pecking one key if the displays were identical and another key if the items in the displays changed in color, orientation, and location. After learning this discrimination, feature binding was tested by swapping one (Experiment 1) or two (Experiment 2) features between items in the display. For example, if the first display contained a red horizontal bar in the upper quadrant and yellow vertical bar in the lower quadrant, then the second display on a swap trial could contain a yellow horizontal bar in the upper quadrant and a red vertical bar in the lower quadrant. If pigeons had bound the features (color, orientation, and location) of the first display, then they should notice that the compound relations among these features in the second display had changed, and thus, should select the key associated with a display change. Pigeons did not respond to these swap trials as they did to change trials, and instead responded to them as if they were identical displays. Thus, pigeons failed to show evidence of feature binding. Human participants learning the same procedure did show evidence of feature binding at test.

Perhaps the task was ill suited to promoting feature binding in pigeons because binding of feature information was not necessary for accurate performance in the task. While all three features were changed on display-change trials, pigeons could have solved the task by attending to only one of the three features. Thus, the ability to use a single feature to solve the discrimination may have prevented feature binding.

The other prior study to look at binding in pigeons used a procedure designed to measure binding errors (Katz et al., 2010). Pigeons learned to detect a target compound when presented with a nontarget compound within the same trial. The compounds were presented simultaneously on some trials and sequentially on others. Trials on which neither compound was a target were included to test for binding errors. For example, if the target compounds were a red ‘U’ shape and a yellow ‘T’ shape, then a test trial might consist of the sequential presentation of a yellow ‘U’ shape followed by a red ‘T’ shape. If pigeons responded to these nontarget compounds similarly to how they respond to the targets, this would be scored as a binding error. Such an error indicates that the pigeon had formed an illusory conjunction of target color with target shape (Treisman, 1996). Some support for binding errors was found in some of the subjects during the initial stage of training but disappeared by the end of training. Binding errors were mostly found in the sequential presentation of nontarget compounds, and very little on trials on which the nontarget compounds were presented simultaneously. The complexity of the task, which involved mixed training on single-item and multiple-item discriminations, and the nuanced phenomena that emerged as a result, such as recency effects and the transient nature of binding effects mitigate the strength of evidence this study can provide for binding in pigeons.

The prior procedures with pigeons utilized tasks of perceptual feature binding and met with limited success. Perhaps a different approach, using a task of memory feature binding would prove more successful. We developed a procedure using an SRT procedure to encourage feature binding in pigeons. In an SRT procedure, items are presented one at a time in rapid succession. The participant is required to respond to each successively presented stimulus as quickly as possible. SRT procedures have a long history of use in the study of implicit and procedural learning in humans (Nissen & Bullemer, 1987) and animals (Christie & Dalrymple-Alford, 2004; Froehlich et al., 2004). Sequence learning in SRT tasks is thought to involve multiple brain regions, from sensory to memory to motor (Keele et al., 2003; Robertson, 2007). In humans, SRT tasks have also been used to study whether object identity, location, or both can be learned. In studies of contextual cueing by which object identity or location can be repeated in sequence, Endo and Takeda (Endo & Takeda, 2004; see also Higuchi et al., 2016; Jiang & Song, 2005) found evidence that each cue can be learned on its own, but when combined, only the more salient cue—typically the cue that is more task relevant—is learned. Similar studies using cues from different modalities to study multisensory-integration have found synergistic effects of having both visual and tactile or visual and auditory cues. Moreover, multisensory congruency supports explicit awareness of sequence information (Silva et al., 2017). Thus, while there is conflicting information as to whether having multiple cues would aid in sequence learning, the SRT procedure provides a plausible alternative to the perceptual binding tasks described above for the study of memory binding in the pigeon.

As far as the ethology of the pigeon is concerned, it could be argued to be functional for the pigeon to bind separate perceptual features, such as the visual properties of an object with its location, if both features have a history of occurring together. For example, millet has a distinctive appearance and is most likely to be found on the ground in a patch of millet grass, whereas black sunflower seeds, which have very different visual features, are most likely found on the ground underneath sunflower plants. Foraging would be optimized by forming associations between objects and their typical locations, thereby allowing spatial locations to elicit search images of likely food, increasing the detectability of food in its typical context (e.g., Kono et al., 1998). Binding involves associative processes, such as Pavlovian conditioning. The functional nature of Pavlovian conditioning (in the evolutionary sense) has been well documented by Domjan and others (e.g., Domjan, 2005). It is still an open question whether properties of visual stimuli that cooccur in time and space gain privileged access to associative processes to promote feature binding, or if they do so despite their arbitrary nature (cf. Domjan et al., 2004). Nevertheless, given the vast evidence for binding of visual features of digital objects in humans, the search for binding in other species is worthwhile and can shed light on comparative questions about the object learning.

The task was simple and was designed to directly manipulate attention to feature conjunctions. In each session, a repeating sequence of four objects (A, B, C, & D) were consecutively displayed in one of four locations (1, 2, 3, & 4) on a touchscreen. The first peck to the object removed it from the screen and the next object was displayed. Reinforcement was periodically delivered following an object peck. Thus, all the pigeon needed to do to earn reinforcement was to peck at an object as soon as it appeared. There were 12 birds placed on this task, and each bird went through two rounds of training. In each round of training, a different set of four objects was used (Fig. 1) and each pigeon was assigned to one of four treatment groups (see Fig. 2 for the four 4-object sequence conditions used in each round). In Group Location-Only Training, the location at which an item appeared repeated in each sequence, but the objects appeared in random order across sequences. In Group Object-Only Training, the objects were presented in a consistent sequence, but the locations at which they appeared were randomized across sequences. In Group Both Training, both object and location appeared in a repeating consistent sequence (e.g., arrow in upper left, shield in upper right, circle in lower left, arrow in lower right). Finally, in Group Random Training, both the objects and locations appeared in randomized sequences.

**Fig. 1:**
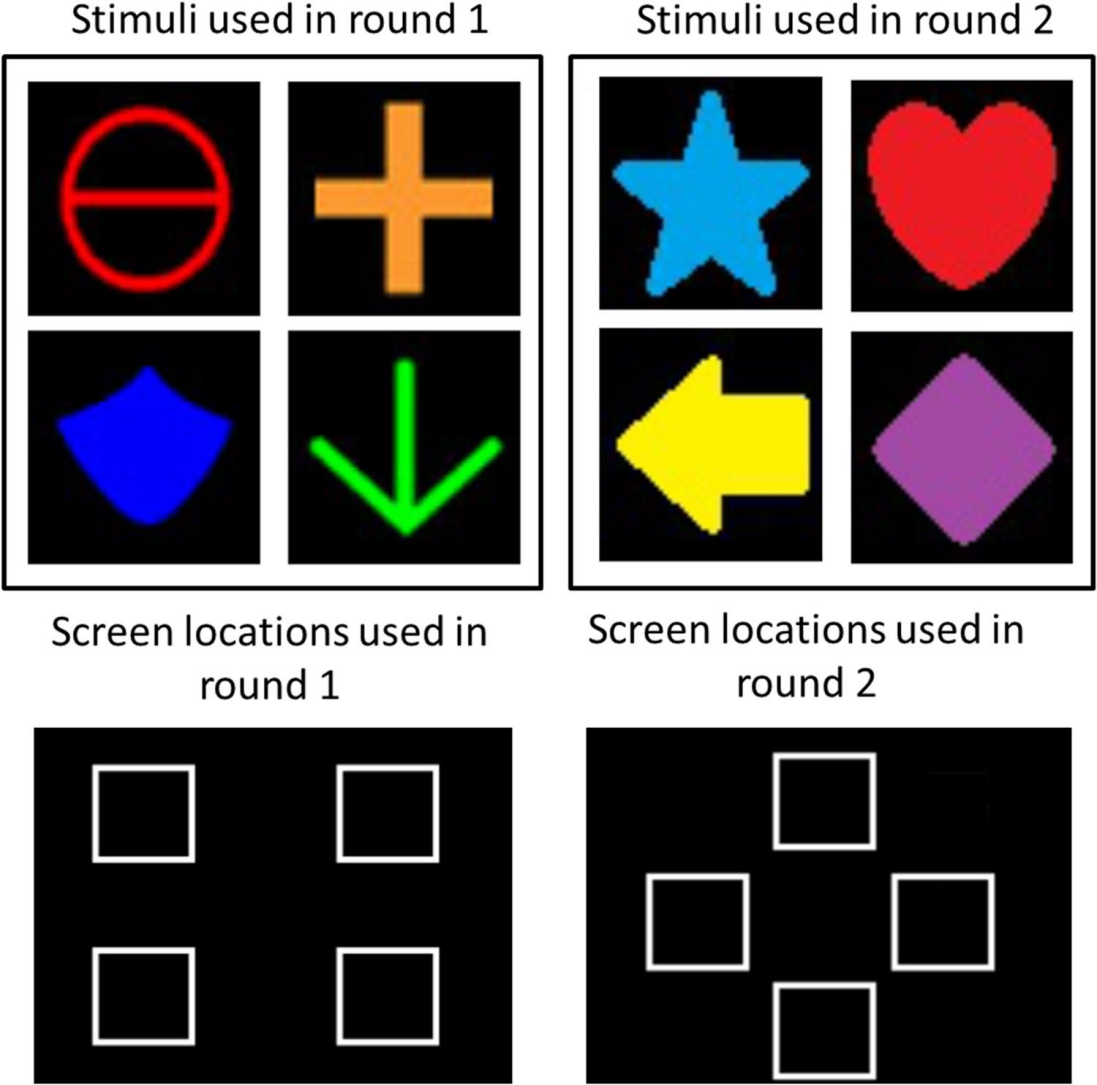
Stimulus sets and screen response locations used in Experiments 1 and 2. Left panels show stimuli and screen locations for round 1 of Experiment 1, and Experiment 2. Right panels show stimuli and screen locations for round 2 of Experiment 1

**Fig. 2:**
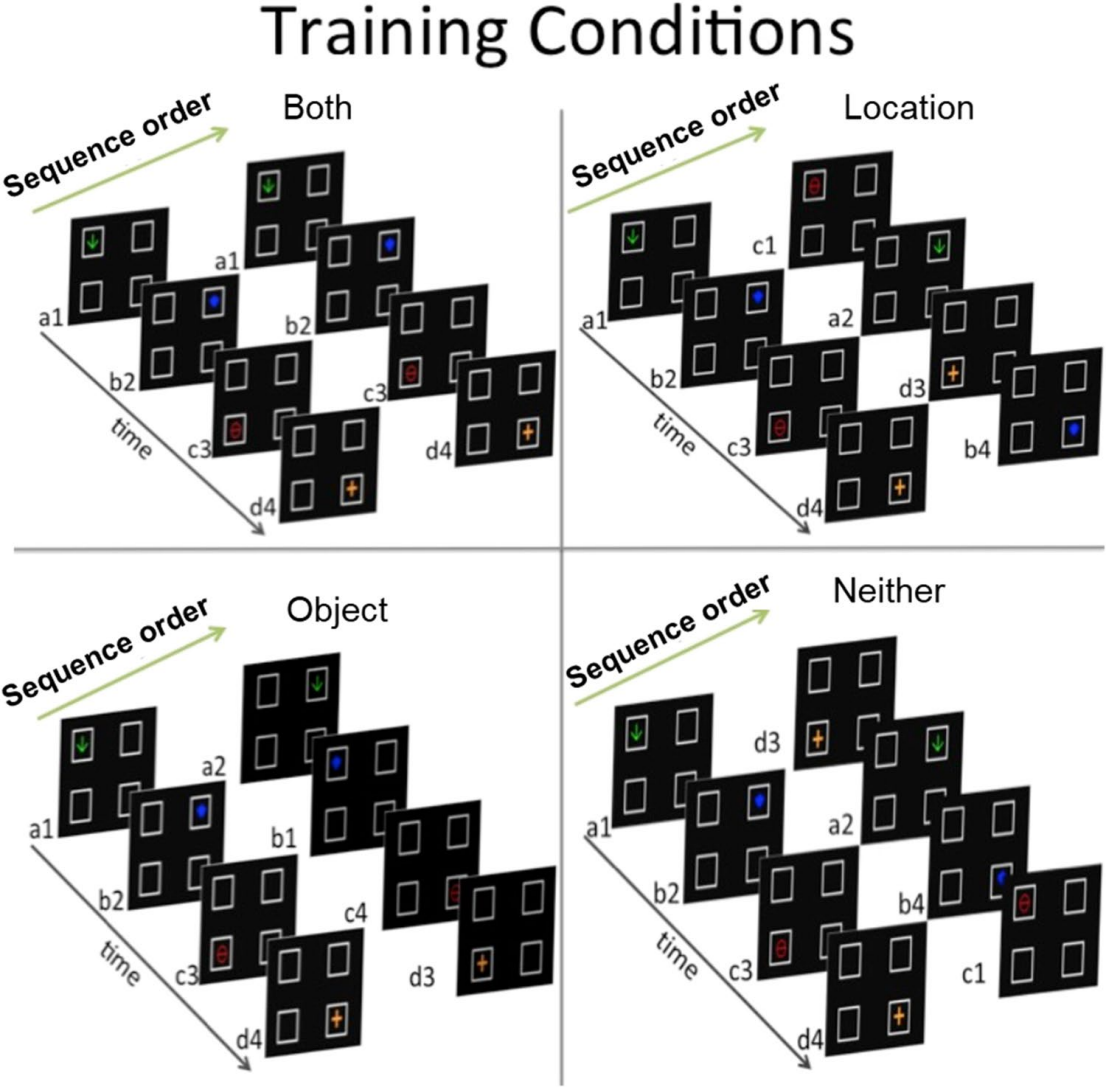
Examples of sequences used in training conditions in Experiment 1. Two consecutive four-item sequences are shown for Group Both Training (top left), Location-Only Training (top right), Object-Only Training (bottom left), and Random Training (bottom right). Each trial consisted of the presentation of a single item at one of the four locations as shown in the displays. In the example for Group Both, the first trial was the presentation of the downward arrow in the upper left location, followed by the presentation of the shield in the upper right quadrant on Trial 2, circle with horizontal line in the lower left quadrant on Trial 3, the plus in the lower right quadrant on Trial 4, the downward arrow in the upper left quadrant on Trial 5 (now in Sequence 2), and so on until the end of the session. Each item remains on the screen until the pigeon makes a single peck to the item

What did we hope to accomplish with this sequence learning procedure? It is well known that, like humans, pigeons are sensitive to repeated sequences which they can readily learn (Froehlich et al., 2004; Herbranson & Stanton, 2011) and can readily learn regularities in sequence structure such as those that underlie artificial grammars (Herbranson & Shimp, 2008). We hypothesized that by repeating sequences, the pigeons in our study should attend to the serial order of information in the repeated sequences. When serial order consisted of repeated locations, pigeons should attend to location. When it consisted of objects, they should attend to object identity. When the order of both object and location were repeated consistently, then pigeons should attend to both. We additionally hypothesize that consistently repeating object and location information together during training should increase attention to both features, and thereby promote memory feature binding. That is, the pigeons should form object–location associations. Alternatively, if one of the features, such as location, is more salient than the other, such as object, as has been found in human studies (Endo & Takeda, 2004; Higuchi et al., 2016; Jiang & Song, 2005), then we might hypothesize that the same overshadowing of object information by location information will be observed in pigeons, in which case memory binding based on object–location associations should be prevented.

To test for sequence learning in Groups Location-Only Training and Object-Only Training, and object–location binding in memory in Group Both Training, nonreinforced probe sequences were delivered. There were four types of probe sequence (Figure 3). Location probes switched the order of locations between two of the four displays within the sequence. Object probes swapped the order of objects between two of the four displays. The remaining two types of probe sequence involved swapping both object and location between two of the displays within the sequence. For Unbound probes, this involved presenting one of the objects at a different location than typical in a consistent sequence condition (see lower right panel of Fig. 3 for an example). This swap resulted in the breaking of any prior object–location association for Group Both Training. Finally, on Bound probes the order of two of the displays were swapped within-sequence (see upper left panel of Figure 3 for an example). By swapping the order of displays, the object–location association was preserved, though now presented out of sequence.

**Fig. 3:**
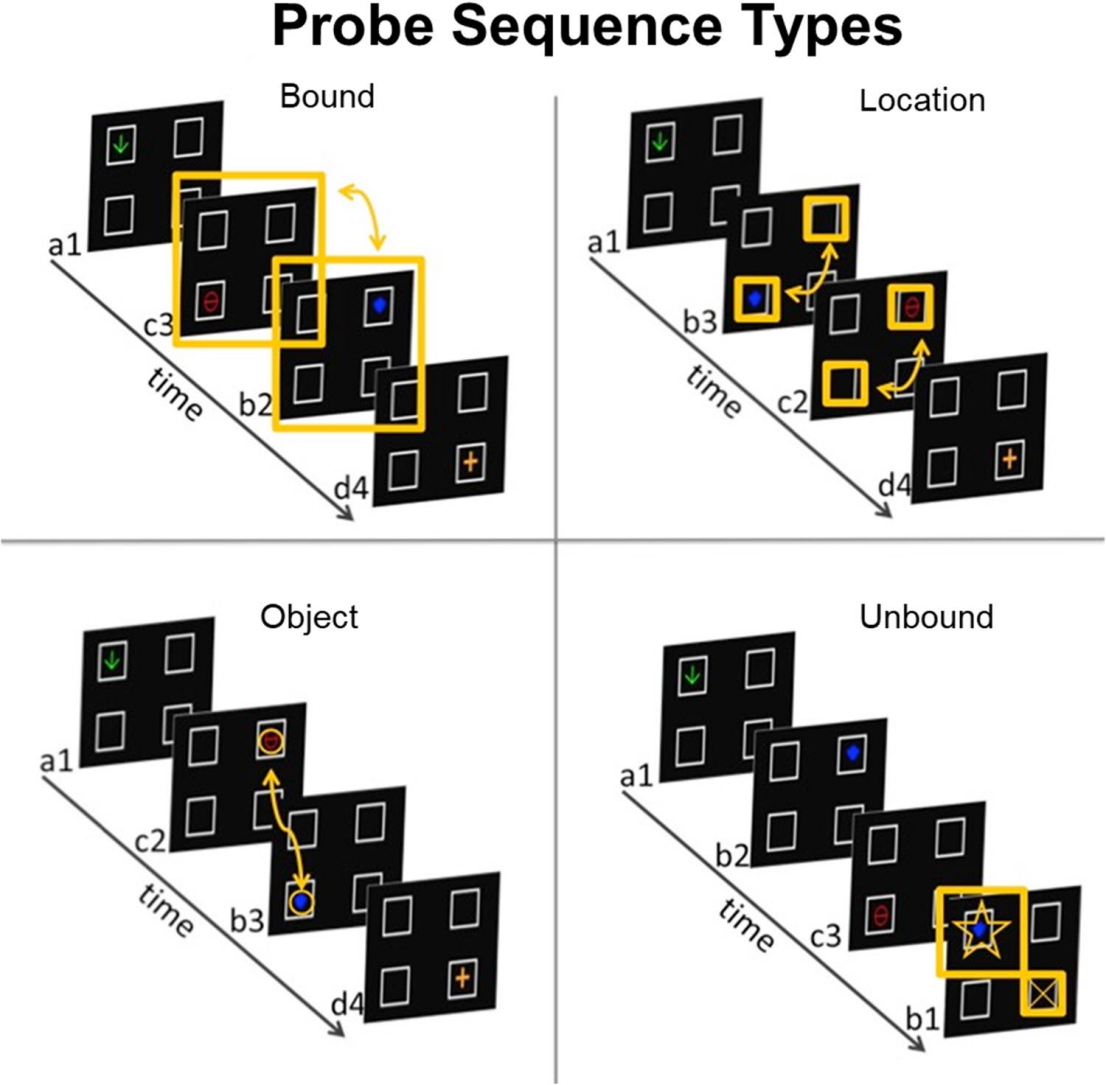
Examples of probe sequences used in testing in Experiments 1 and 2. In Bound probe sequences (top left), the order of two of the displays were swapped. In Location probe sequences (top right), the order of two locations were switched. In Object probe sequences (bottom left), the order of two of the objects were switched. In Unbound probe sequences (lower right), one of the items in the sequence consisted of a repeat of an object and location already used in that sequence such that the object–location association differed from that used in the training sequence

The probe sequences systematically broke sequence consistency of object, location, or both for subjects that had received those features in a consistent sequence during training. Thus, if pigeons in Group Both Training had bound object–location information during training, as hypothesized above, then any change that disrupts object–location binding should result in slower response times (RTs) to these displays compared with training displays in which object–location information is preserved. That is, there should be an RT cost on probe sequences if binding is violated, but only for pigeons that had bound object and location information. The object–location relationship is broken on Object, Location, and Unbound probes. Only the Bound probes preserves the object–location association, and thus we predicted much less RT cost on Bound probes by birds in Group Both Training. RT cost was measured as a percent increase in mean RT on probe sequences relative to training (baseline) sequences.

Additional predictions can be made about pigeons in the other three treatment groups. The order of locations was consistent during training for Group Location-Only Training, therefore switching the order on Location and Bound probes should induce an RT cost for those birds, while switching the order of objects should not. Likewise, switching object order on Object and Bound probes should induce an RT cost for pigeons trained on object consistent displays (Group Object-Only Training), while no RT cost should be observed on Location switch probes. Finally, pigeons in Group Random Training experienced no consistency in sequence information during training, and thus were not expected to show any RT costs on any probe sequence.

## Experiment 1

In Experiment 1, pigeons were consecutively trained on two 4-item sequences that repeated throughout each session: Location consistent, Object consistent, Both consistent, or Random. Each of the two sequences were trained in separate rounds of training and testing (described below). Once responding had stabilized at asymptotic rates, probe sequences were presented. Probe sequences could disrupt various aspects of sequence information, such as location, object, or both.

### Methods

#### Subjects

Twelve pigeons (*Columba livia*) served as subjects. They had previously participated in a variety of open-field and touchscreen experiments but were naïve with respect to the stimuli and sequence-learning task used in this experiment. Pigeons were maintained at 80%–85% of their free-feeding weights and were individually housed in a colony with a 12-h light–dark cycle. They had free access to water and grit in their home cage. Experimental procedures occurred during the light portion of the cycle.

#### Apparatus

Training and testing were conducted in a flat-black Plexiglas chamber (38 cm wide × 36 cm deep × 38 cm high). All stimuli were presented by computer on a color LCD monitor (NEC MultiSync LCD1550M) visible through a 23.2 cm × 30.5 cm viewing window in the middle of the front panel of the chamber. The bottom edge of the viewing window was 13 cm above the chamber floor. Pecks to the monitor were detected by an infrared touchscreen (Carroll Touch, Elotouch Systems, Fremont, CA) mounted on the front panel. A 28-V houselight located in the ceiling of the box was illuminated at all times, except when an incorrect choice was made. A servo-driven food hopper (produced in house) was located behind the front panel with its access hole flush with the floor centered below the touchscreen. All experimental events were controlled and recorded with a Pentium III-class computer (Dell, Austin, TX, USA). A video card controlled the monitor in the SVGA graphics mode (800 pixels × 600 pixels). The stimuli used in this procedure are similar to those used by (Blaisdell & Cook, 2005). The stimuli and 3 x 3-cm screen locations where they could be presented are shown in Fig. 1 separately for each round of testing. For Round 1, these locations were arranged 8 cm from the top, 7 cm from the sides, and 4 cm from the bottom of the screen. For Round 2, these locations were arranged by rotating the 2 × 2 grid of response boxes by 45 degrees such that one location was centered horizontally along the upper part of the screen, another centered horizontally along the lower part, and the remaining two centered vertically along the left and right sides of the screen. Each response box was surrounded by a white border to demark the location where the stimuli could appear.

#### Procedure

Pigeons had previously been trained to eat grain from the food hopper (magazine training), so training began immediately with a mixed autoshaping/instrumental shaping procedure using the sequence-learning task. Each trial consisted of the presentation of one of four objects (A, B, C, & D) at one of four locations (1, 2, 3, & 4) on the screen. Each bird was randomly assigned to one of four conditions in which presentation order could occur in a repeating sequence. For birds in Group Location-Only Training, the locations were ordered in a repeating sequence (1 ➔ 2 ➔ 3 ➔ 4) but object order was randomized in blocks of four trials (Fig. 2, top-right panel). For birds in Group Object-Only Training, the objects were presented in a recurring sequence (A ➔ B ➔ C ➔ D), but location order was randomized in blocks of four trials (Fig. 2, bottom-left panel). Items were presented on the screen in one of the array locations, one at a time, and remained on-screen for either 30 s or until pecked. Once the 30 s had elapsed or the item had been pecked, whichever came first, the item was removed, and the hopper was raised for 3 s. As the hopper was lowered, the next item was presented. The object and location of each item depended on treatment group. For subjects in Group Location-Only Training, the locations where objects could be presented were presented in a consistent, repeating order, but the order of objects was randomized across sequences (e.g., A1, B2, C3, D4, C1, A2, D3, C4). For subjects in Group Object-Only Training, the objects were presented in a consistent, repeating order, but location order was randomized across sequences (e.g., A1, B2, C3, D4, A3, B4, C1, D2). For subjects in Group Both Training, both object and location order were consistent and repeated across sequences (e.g., A1, B2, C3, D4, A1, B2, C3, D4). For subjects in Group Random Training, the location and object order were randomized (e.g., A1, B2, C3, D4, B3, A4, D2, C1). Randomization was done by scrambling the four objects and/or locations for each four-item sequence.

Once subjects were responding on a majority of trials (>80%), the 30-s trial time limit was discontinued, and the item remained on screen until pecked. During this phase the interstimulus interval (ISI) was randomly selected from a range between 5 and 15 s. As peck rate increased, the ratio of rewarded to unrewarded trials was decreased until reinforcement was delivered on average every 20th trial. Any trial that followed a rewarded trial was excluded from analysis to avoid artefacts introduced by the consummatory response. Test sessions began once peck rates had stabilized.

Test sessions were identical to training sessions, except that 20 of the 90 training sequences were replaced with nonreinforced probe sequences. Four types of probe sequence were used for all subjects regardless of their training condition (see an example of each in Fig. 3). Each probe sequence consisted of a change in the four-item sequence. On Object probe sequences, the order of two of the objects in the sequence were swapped with each other without altering the sequence of locations. On Location probe sequences, the order of two locations in the sequence were swapped without altering the sequence of objects. On bound probe sequences, the order of two objects and locations were swapped such that the object–location association that occurred during training was maintained (e.g., instead of A1, B2, C3, D4, the subject might receive A1, **C3**, **B2**, D4, with the boldface indicating the swapped items). On Unbound sequences, one of the items in the sequence consisted of a repeat of an object and location used elsewhere in the sequence such that the object–location association differed from that used in the training sequence (e.g., instead of A1, B2, C3, D4, the subject might receive A1, **D3**, C3, D4, with the boldface indicating the probe item). At least three training sequences occurred before a probe sequence was scheduled for delivery, and the first probe sequence was not presented until after the first 10 training sequences in the test session had occurred.

After collecting probe data in Round 1, pigeons were reassigned to new treatment groups in counterbalanced fashion, such that one bird from each treatment condition was assigned to one of the other treatment conditions (e.g., for Group Both, one bird was reassigned to Group Location, another to Group Object, and the third to Group Random Training). After reassignment, birds received a new round of training and testing using the same procedures as for Round 1, with the exception that new stimuli and screen locations were used (Fig. 1, right panel). RT from stimulus onset to subject peck in the response box was recorded on each trial and served as our dependent measure.

#### Data analysis

Baseline and Probe sequence data were collapsed across all sessions for the last 100 probe sequences, separately for each subject and for each replication, resulting in a final *n* = 6 per treatment group. RT cost on probe sequences was normalized for each subject by subtracting mean Baseline RT of the preceding baseline sequence from mean Probe sequence RT on the subsequent probe sequence, and this difference was then divided by mean Baseline RT and multiplied by 100 to yield a percentage change in RT. For example, if the mean Baseline RT was 1 s and the mean Probe RT was 1.5 s, RT cost would be calculated as (1.5 − 1)/1 = .5 × 100 = 50%.

While mean Probe sequence RTs were compared with mean RTs of the preceding baseline sequences, there were a few restrictions placed on baseline RT selection for this calculation. Mean baseline RTs were only taken from baseline sequences in which reinforcement had not been delivered. Data from any baseline and probe sequence on which the first response was outside the response box in which the stimulus was present were excluded from analysis.

### Results and discussion

**Fig. 4:**
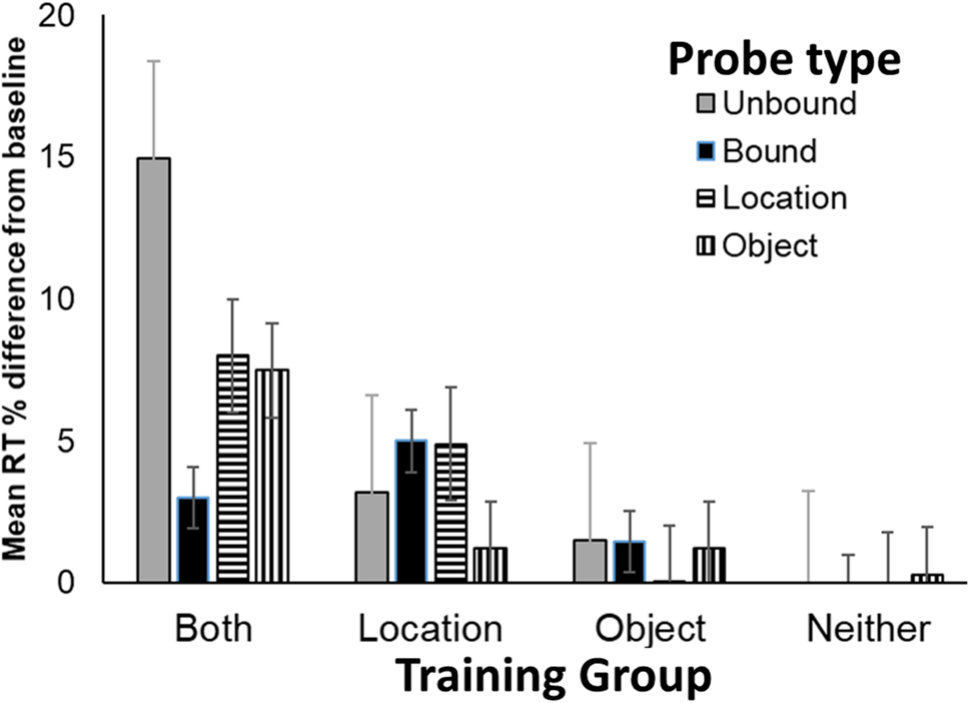
Mean Response Time (RT) cost in seconds for each probe sequence type (Unbound, Bound, Location, and Object) shown separately for Groups Both Training, Location-Only Training, Object-Only Training, and Random Training in Experiment 1. Error bars depict mean standard errors

Training required an average of 103 (range = 96–115) sessions to reach training criterion. By the end of training, RT on baseline trials was a mean +/- *SEM* of 1.05 +/- 0.06 s and remained unchanged during test sessions. A mixed analysis of variance (ANOVA) conducted on the percentage difference RT, with Group as a between-group factor and Probe Type as a repeated measure, revealed main effects of Group, *F*(3, 20) = 7.80, *p* < .01, and Probe Type, *F*(3, 60) = 3.17, *p* < .05, as well as a Group × Probe Type interaction, *F*(9, 60) = 5.24, *p* < .01 (Fig. 4). Planned comparisons were performed to test our specific predictions. In Group Location-Only Training, RT cost on object probe sequences was lower than on the other three probe sequences where location was changed, smallest *F*(9, 20) = 9.02, *p* < .01. In Group Both Training, RT cost was higher on Unbound probe sequences than on all other probe sequences, smallest *F*(1, 20) = 15.90, *p* < .01. Furthermore, RT cost was lower on Bound probe sequences than all other probe sequences, smallest *F*(1, 20) = 11.16, *p* < .01. These results support the hypothesis that pigeons in Group Both Training, that had received training with both location and object in a consistent sequence, had perceptually bound object and location information of the items. No significant differences were found between probe types in Group Object-Only Training or Group Random Training, *F*s(1, 20) < 1.0.

Group Random Training failed to show any RT costs on probe trials. Thus, this group serves as a useful control in that no RT costs were found under training and testing conditions in which they were not expected. No effects were predicted because sequence order during training was random for both object and location information. Switching the order on probe trials was undetectable because order was already random during training.

For Group Object-Only Training, only the order of objects was consistent during training, so we predicted that switching object order on Object probes should produce an RT cost. No RT cost was observed, however, which suggests that birds in this group did NOT encode the order of object during training.

For Group Location-Only Training, location order was encoded as evidence by the higher RT costs on probe types on which location order was changed. Thus, the failure for pigeons in Group Object-Only Training to encode object order was not due to fundamental limitations with the procedure itself. Instead, it likely reflects object information being less salient than location information. In retrospect this makes sense because responding is motivated by food. Thus, any predictive information that could facilitate rapid responding should receive greater attention. Location information does provide predictive information as to where the next item in the list will appear, whereas object information cannot be used to reduce response times because all the subject need do is peck at an item as soon as it appears. The identity of the object is irrelevant.

Nevertheless, in probes in which only the order of objects was changed (Object probe sequences), RT increased only for pigeons in Group Both Training. Group Both Training is the only group for which object–location information was correlated during training. The increased RT on object probe sequences in Group Both Training suggests that the object and location had become perceptually bound during training, such that any probe sequence that broke this association resulted in an RT cost. Correlating object and location consistency had the benefit of increasing the salience of object order information, probably due its being bound to location information which pigeons did encode even in the absence of a consistent object order (i.e., in Group Location-Only Training). The sequence learning procedure therefore appears to have enabled recruitment of attentional processing of location information. Furthermore, when object information was correlated with location information, the increased attention to object order enabled object–location feature binding similar to what has been reported in primates.

## Experiment 2

The primary results of Experiment 1 were (a) the finding that birds encoded location order when it was consistent within sequence (b) but failed to encode object order when it was consistent within sequence; (c) when both object and location order were consistent within sequence, then pigeons encoded both object and location order; and (d) consistently repeating object and location order together within sequence resulted in object–location binding.

**Fig. 5:**
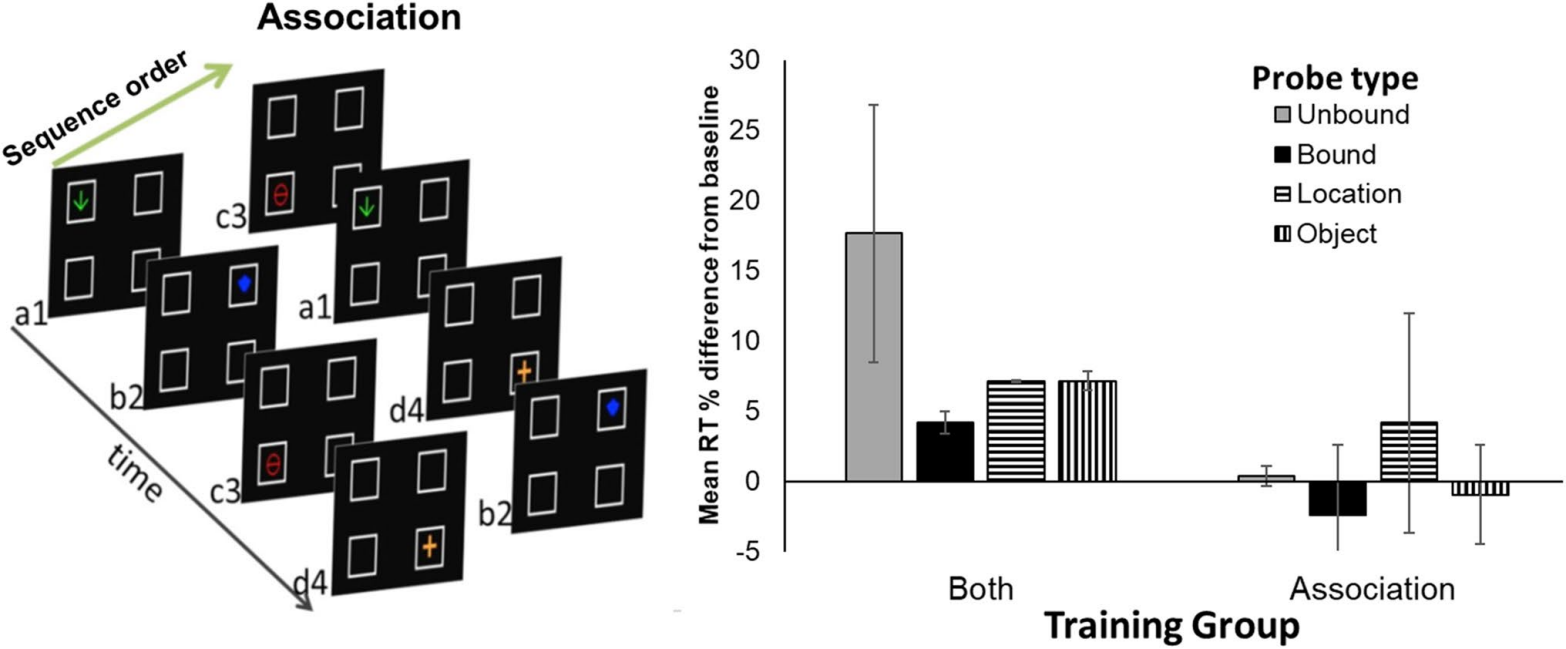
Left panel: Examples of sequences used in training condition for Group Association in Experiment 2. Right panel: Mean Response Time (RT) cost in seconds for each probe sequence type (Unbound, Bound, Location, and Object) shown separately for Groups Both Training and Association in Experiment 2. Error bars depict mean standard errors

By ordering both location and object in a consistent sequence, the same object always appeared at the same location. This could enable the establishment of object–location associations. While the association between a location and an object may be learned through such repeated pairings, it was not clear if such pairings were sufficient for learning of object–location associations. The sequential aspect to this task offered many additional cues to increase the salience of the object–location pairings, such as local statistical information between adjacent items (Froehlich et al., 2004). In the study by Froehlich et al. (2004), using a serial reaction time task, response times were faster to repeated lists compared with randomly ordered item lists. This shows that pigeons benefit from item repetition within lists to facilitate attention to the next anticipated item. In Experiment 2, we address the following question: Was having consistent object–location correlation sufficient for binding, or was sequence consistency also necessary for object–location binding? To answer this question, we trained six new pigeons on the sequence-learning procedure. Three of the pigeons received the same training as Group Both Training received in the first round of Experiment 1. The other three pigeons also received training on stimuli for which the same object always appeared at the same location but presentation order was randomized across sequences (Fig. 5, left panel); thus, we call this treatment group the Association condition because it could allow for the establishment of an object–place association despite randomization of within-sequence order. After peck rates had stabilized, pigeons received the same series of probe tests as described in Experiment 1. If consistency between object and location was sufficient to support feature binding, then Groups Both Training and Association should both show the same pattern of RT cost effects on probe trials as was shown for Group Both Training in Experiment 1. Alternatively, if maintaining a consistent sequence for both object and location is necessary for binding, then we only expect to replicate the pattern of RT cost effects in Group Both Training, but not in Group Association.

### Methods

#### Subjects

The subjects were six new pigeons (*Columba livia*) from the same colony as those used in Experiment 1, but naïve with respect to the current experimental stimuli and procedure.

#### Apparatus

The apparatus was the same as in Experiment 1.

#### Procedure

Pigeons were randomly assigned to one of two groups—Both Training and Association. Training proceeded as in Experiment 1 using the stimuli and locations used in round 1 (Fig. 1, left panels). Group Both Training received the same treatment as described for Group Both Training in Round 1 of Experiment 1. Group Association received training on a similar procedure as Group Both Training in which each object could only appear at a specific location with the exception that item order was randomized within-sequence (e.g., A1, B2, C3, D4, B2, D4, A1, C3; Fig. 5, left panel).

### Results and discussion

Inspection of individual bird data revealed that two of the pigeons in Group Both Training replicated the pattern of results from Experiment 1, while a third pigeon did not replicate this pattern of results and showed no evidence of binding, despite training for Group Both Training. Thus, data from this nonreplicating subject were excluded from analysis.

Training required an average of 97 (range: 95–103) sessions to reach training criterion. By the end of training, RT on baseline trials was a mean +/- *SEM* of 1.02 +/- 0.12 s and remained unchanged during test sessions. A mixed ANOVA conducted on the percentage difference RT, with Group as a between-group factor and Probe Type as a repeated measure, revealed an effect of Group, *F*(1, 3) = 128.15, *p* < .01, but no effect of Probe Type, *F*(3, 9) = 2.74, *p* > .10, nor a Group × Probe Type interaction, *F*(3, 9) = 2.08, *p* > .10 (Fig. 5, right panel). Note, with only two pigeons in Group Both and three pigeons in Group Association, the experiment was likely underpowered to detect the interaction between factors. Due to the smaller sample size in Experiment 2 than Experiment 1, we only report post hoc Tukey tests to assess differences. The only significant pair wise comparison was the slower RTs to Unbound probes in Group Both than in Group Association. Probably due to a low sample size, we failed to replicate the difference between the Unbound and Bound probe tests in Group Both.

We qualitatively replicated the pattern of differences between probe conditions found in Group Both Training in Experiment 1. Some of the replication failures likely result from the small *n* in Group Both Training in Experiment 2. For the new condition, Group Association, none of the probe trials differed from each other. In particular, the lack of a difference in RT cost on probe trials that preserved the object–location association (Bound probes) versus those on which the object–location association was broken (Unbound probes), and that there was no RT cost on either of these probe sequences suggests that the pigeons in this treatment group did not bind object and location information during training. Thus, it appears that merely correlating object and location information was not sufficient for memory feature binding, and that having a consistent sequence during training for both object and location information was necessary for object–location binding to occur. These results point to the importance of local statistical information in driving attentional processes necessary for feature binding (Froehlich et al., 2004).

Sequence consistency in Group Both Training may have facilitated object–location binding in several ways. Since location order was consistent, pigeons could predict where each next object would appear based on the location of the previous object. This could allow the pigeon to begin attending to the anticipated location prior to the onset of the object. As a result, when the object finally appears, the pigeon is better prepared to process that object information. Increased processing of the object prior to its being pecked (and thus disappearing from the screen) could facilitate object–location binding. Since the location of each object could not be anticipated by pigeons in Group Association, objects did not benefit from this extra processing, and thus failed to be bound with location information, despite the fact that each object could only appear (and thus be associated with) a particular location. Processing of visual information has been shown to similarly benefit from other techniques that increase anticipation of information, such as priming in visual search (D. S. Blough, 2000; P. M. Blough, 1989; Bond & Kamil, 1999; Cook et al., 1997).

## General discussion

Pigeons were presented with a repeated sequence of four objects at four locations. For different groups of pigeons in Experiment 1, the objects, locations, or both could be repeated in a consistent or random order. To test for encoding of ordered sequences, probe tests were conducted in which object order, location order, or both could be disrupted. These tests revealed that pigeons had encoded the location order in both Group Location-Only Training and Group Both Training. There was no evidence that object order was encoded in Group Object-Only Training, but object order was encoded in Group Both Training. It is possible that object order was encoded by birds in Group Object-Only Training, but they might not have been able to use this knowledge to prepare their response to the next item to be presented. Knowledge of upcoming objects is less likely to produce robust RT facilitation than knowledge of upcoming locations, the latter allowing for preparatory responding. Moreover, for pigeons in Group Both Training, changing the object and location order such that the object–order relationship used in training was preserved had much less impact, evidenced by low RT costs, compared with changing the object and location order such that the object–location relationship used in training was broken. This difference reflects object–location binding by pigeons in Group Both Training. In Experiment 2, when object–location association was maintained, but sequence order randomized (Group Association), no binding was found. Thus, feature binding depended on maintaining a consistent item sequence order. This is consistent with other reports using similar procedures that show that local statistical information facilitates anticipatory responses in pigeons (Froehlich et al., 2004). By facilitating anticipatory responding, attention to feature conjunctions, such as between object and location, is enhanced, thereby facilitating feature binding.

This is the first evidence for strong and lasting memory feature binding in pigeons. Notably, Treisman suggested location binding in which objects are bound to their location may be the most basic binding problem brains are designed to solve (Treisman, 1996). Presenting objects and locations in a consistent order within sequence was necessary for their binding. Prior studies have shown that pigeons will naturally (i.e., without explicit reinforcement) encode sequences of locations (Froehlich et al., 2004; Herbranson et al., 2014; Herbranson & Stanton, 2011), though they can also learn sequences through reinforcement (Garlick et al., 2016). Perhaps prior studies that failed to show lasting feature binding resulted from procedures that failed to sufficiently increase the salience of, and thereby attention to, feature conjunctions.

This is the first report of strong and lasting memory binding in pigeons. Past attempts at identifying object–location binding in pigeons have used very different procedures. Lazareva and Wasserman (2016) found no evidence of object–location binding in pigeons using a change detection task. Their procedure used presentations of displays made from multiple colored lines at varying orientations. Though birds learned to classify displays as different if they contained novel orientations or colors, trials in which only one or two of the three properties were altered between slides were treated as being the same. This was taken as evidence that pigeons did not bind feature information about the objects. Nevertheless, as discussed in the Introduction, as they were trained, pigeons could easily solve their task by attending to only one feature at a time. There was no need to attend to multiple dimensions simultaneously. Binding appears to require attention to multiple features so that they can be integrated into a configural object representation. Likewise, the positive evidence for feature binding in pigeons reported by Katz et al. (2010) was fleeting and not found in all pigeons tested. Thus, while their procedure may have initially caused the pigeons to attend to feature conjunctions, behavioral control by these conjunctions waned with further training.

Even in our procedure, reinforcement did not depend on object–location encoding, and thus the same criticism could be directed here. Nevertheless, pigeons trained in Group Both Training, where items with consistent object–location associations were presented in a consistent sequence, did show evidence of object–location memory binding, thus obviating this criticism. Apparently, something about our procedure worked, where others have failed or met with limited success. Perhaps it had to do with the fact that performance was facilitated by attending to location information. As a result, conjunctions between object and location features (Experiments 1 and 2, Group Both Training) may have increased in salience compared with conditions in which object–location conjunctions were present, but location order was random (Experiment 2, Group Association).

These experiments add to the growing literature on the pigeon visual system. The architecture of the higher level pallial structures of the avian and mammalian brain differ markedly. Like other classes of vertebrates, the avian pallium is organized as a network of nucleated circuits. The mammalian pallium, however, consists of a laminar isocortex. Nevertheless, despite these architectural differences, the functional connectivity of bird and mammal pallium are quite similar Reiner et al. (2004). Thus, the avian brain is a useful animal model for studying human cognitive neuroscience (Clayton & Emery, 2015). Furthermore, like the mammalian visual system, the avian visual system is adapted to the rapid processing of visual input (Cook, 2000) and thus similarities in mammalian and avian visual systems can be anticipated on functional grounds. This involves the operation of similar mechanisms of early vision and search, such as the segregation of visual input through separate channels, and their recombination at higher order brain areas (Wylie et al., 2009). For example, features such as color, shape, and size have been shown to be processed independently (Cook, 1992b; Cook et al., 1996) and reintegrated to meet task demands such as visual search for targets defined by specific feature conjunctions (Cook et al., 1996; George & Pearce, 2003). The ability to discriminate target items based on feature conjunctions is a necessary precursor to feature binding. Feature binding goes beyond feature conjunctions in that binding is automatic and plays a fundamental role in object learning and recognition. By using an implicit learning task involving ordered sequences, our experiments provide strong evidence for the automatic binding of features in pigeons.
